# Plasmon-induced nanoscale quantised conductance filaments

**DOI:** 10.1038/s41598-017-02976-7

**Published:** 2017-06-06

**Authors:** Vasyl G. Kravets, Owen P. Marshall, Fred Schedin, Francisco J. Rodriguez, Alexander A. Zhukov, Ali Gholinia, Eric Prestat, Sarah J. Haigh, Alexander N. Grigorenko

**Affiliations:** 1 School of Physics and Astronomy, University of Manchester, Manchester, M13 9PL UK; 2 National Graphene Institute, University of Manchester, Manchester, M13 9PL UK; 3 School of Materials, University of Manchester, Manchester, M13 9PL UK

## Abstract

Plasmon-induced phenomena have recently attracted considerable attention. At the same time, relatively little research has been conducted on electrochemistry mediated by plasmon excitations. Here we report plasmon-induced formation of nanoscale quantized conductance filaments within metal-insulator-metal heterostructures. Plasmon-enhanced electromagnetic fields in an array of gold nanodots provide a straightforward means of forming conductive CrO_x_ bridges across a thin native chromium oxide barrier between the nanodots and an underlying metallic Cr layer. The existence of these nanoscale conducting filaments is verified by transmission electron microscopy and contact resistance measurements. Their conductance was interrogated optically, revealing quantised relative transmission of light through the heterostructures across a wavelength range of 1–12 μm. Such plasmon-induced electrochemical processes open up new possibilities for the development of scalable devices governed by light.

## Introduction

Due to high field compression and enhancement ratios^1^, plasmonics promise to bring about advances in optics^2^, improve bio-sensing^3^ and optical trapping^4^, and speed-up data communications^5^. Recently, plasmon-induced phenomena^6, 7^, particularly in hybrid plasmonic systems^8^, have attracted considerable attention. Plasmon-induced hot carriers could ensure progress in photovoltaics^6^, fast electronics and photo-chemistry^7^, as well as catalytic reactions^9^ including those required for green energy production^10^. At the same time, relatively little research has been conducted on plasmon-induced electrochemistry which can be very useful for creation of resistive random access memory (RRAM) devices.

RRAM is a promising candidate for the next generation of nonvolatile memory^11–15^. It consists of a simple metal-insulator-metal (MIM) layered structure, and is based upon the principle of controllable switching between different electrical resistance states. Such switching can be achieved through the formation and dissolution of nanoscale conductive filaments (CFs)^13–15^. Among the numerous demonstrations of RRAM nanocells reported to date^11–22^, a great deal of attention has been given to CF-based approaches due to their superior performance characteristics (low electrical switching powers, high speed operation and technological scalability) and easy CMOS integration (simple material systems and structures)^18–22^. A variety of oxide, chalcogenide, and sulphide thin films have been proposed for use as the solid electrolyte core material^13–16^. Application of a sufficient voltage across the electrodes leads to field-assisted injection and transport of cations within the insulator, and hence the growth of a CF and the creation of a memory cell^19–22^. In most cases CFs are detected by recording the resistance state of studied RRAM nanocells, which sometimes exhibit quantised conductance^23^.

**Figure 1:**
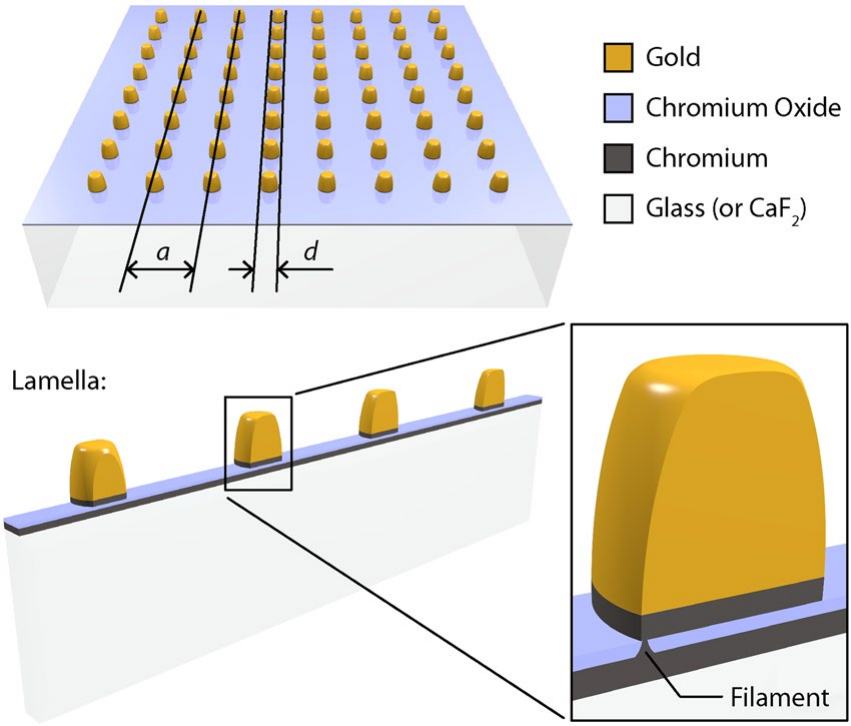
Device schematic. Plasmonic ND arrays were fabricated on glass (or CaF_2_) substrates covered in a thin Cr layer. Thin lamellas, intersecting multiple NDs, were extracted from arrays in order to perform STEM measurement of conductive CrO_x_ filaments.

One area of particular interest is that of all-optical memory devices, where both the writing and reading of information is performed by light. Recently, we found a system which could be a viable candidate for all-optical memory based on CFs. To explain the quantised relative transparency of plasmonic nanoarrays^24^, we suggested that conductive filaments can be formed by light in metal-insulator-metal structures with the help of a simple plasmonic mechanism. In brief, consider a regular array of nanoparticles fabricated on an insulator-metal heterostructure (where both insulator and metal are assumed to be thin), as illustrated in Fig. 1. Light incident on the structure will excite localised plasmon resonances (LPRs) and induce strongly enhanced and localised electromagnetic fields inside the insulator. These fields can be rectified^25^ and can lead to ion transport and chemical reactions^24, 26–28^ in the insulator layer, allowing CFs to grow. As soon as CFs are formed between the metal nanoparticles and metallic sublayer, the shunt currents through the sublayer suppress the LPRs and hence the driving force for further CF growth disappears. This natural feedback loop halts CF growth at the stage when the CF has just one single conducting channel and hence one conductance quantum for sufficiently small dots. The resistance of narrow breakdown/contact channels is known to be quantized^29^. For larger dots or smaller array periods several conducting channels may be needed to suppress LPRs of the nanoparticles. It is worth noting that the suppression of LPRs and diffractive coupled resonances^30, 31^ should involve the physical transfer of electrons between nanodots, which can only occur in the presence of CFs^32–38^. Remarkably, the relative optical absorption of a regular square nanoarray (spectrally far from the LPR) depends only on the average resistance *R* between nanodots and is given by ${Abs}\approx {\rm{Re}}(\frac{4\pi }{cR})$, where *c* is the speed of light^24^. Hence, one can directly probe the conductance of filaments by measuring the relative optical transparency of a sample. This allows optical read-out of the information stored in the system.

Previously, we extensively studied regular square arrays of high quality Au nanodots (NDs), fabricated on top of a thin Cr sublayer and observed quantized relative infrared transmission defined solely by universal constants^24^. We provided several indirect confirmations that this optical quantization stems from a quantized shunt resistance produced in the thin native Cr oxide (Cr_2_O_3_) layer covering the Cr sublayer^24^. The aim of this work is to offer a direct proof for the existence of light-induced CFs in the native Cr oxide layer produced by the plasmonic mechanism described above. Furthermore, we explicitly show that the optical conductance of the CFs reaches a quantized value over an extremely wide wavelength range, as directly assessed from the optical transmission and electrical measurements. In contrast to prior electrical write-optical read devices, in which the transmission of plasmonic waveguides have been altered^39, 40^, our approach combines plasmonic writing with a free space optical readout technique. One can envisage non-volatile non-magnetic carriers of information based on the studied effect. Our work encourages further investigation of quantum plasmonic systems and plasmonic all-optical nanoscale memory.

## Results and Discussion

Au ND arrays (*a*: the lattice constant, *d*: dot diameter) were fabricated on a thin chromium sublayer (see Methods). They were switched to a conducting state — with suppressed LPRs and quantised relative transmission due to CF formation — by natural light illumination.

In order to confirm the existence of CFs in the fabricated heterostructure thin lamellas were extracted from ND arrays, as illustrated in Fig. 1, using an FEI Nova Nanolab 600 focussed ion beam system and a lift-out technique (see Methods). These lamellas were then studied by high resolution STEM imaging and analysis techniques. Specifically, elemental mapping was performed on a number of ND cross-sections using energy dispersive X-ray spectroscopy (EDS) and electron energy loss spectroscopy (EELS) in an FEI Titan G2 ChemiSTEM microscope.

**Figure 2:**
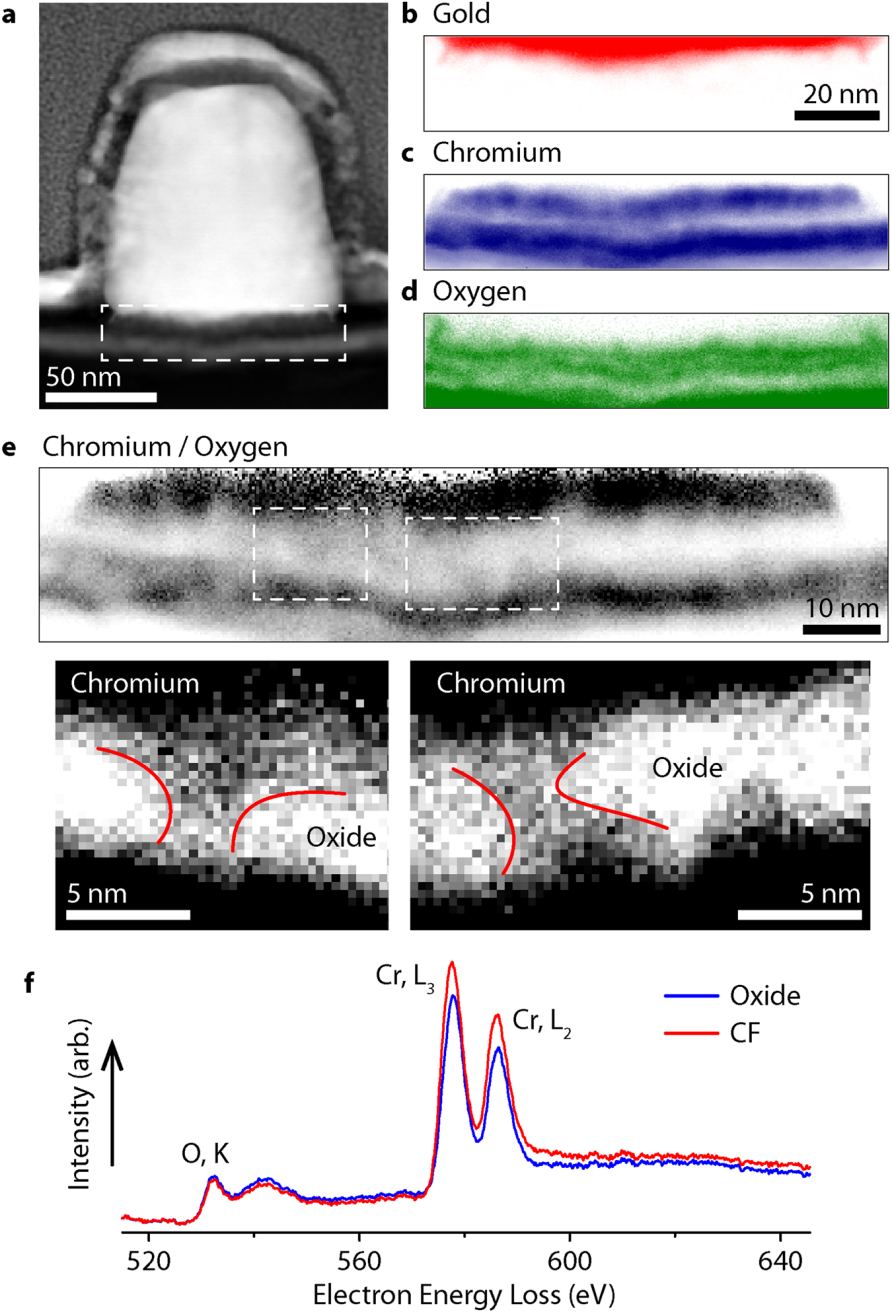
STEM measurement of plasmonically-formed conductive filaments in Cr oxide. (**a**) STEM HAADF image of a typical nanodot cross section. Note the sample was covered in protective layers during lamella fabrication (the visible interface is a result of a varying deposition method and rate). (**b**–**d**) Elemental distribution maps for Au (EDS, M edge), Cr (EELS, L edge) and O (EELS, K edge), within the highlighted region of (**a**). (**e**) Relative Cr/O ratio map (darker areas show higher relative Cr concentration). Expanded regions, with enhanced map contrast, reveal filaments of elevated Cr content spanning the oxide layer (lines added as an aid to the eye). (**f**) Typical EELs spectra from the oxide and CF regions.

A SEM image of a typical ND cross-section is presented in Fig. 2(a), whereas Fig. 2(b–d) show elemental distribution maps for Au (red), Cr (blue) and O (green) within the dashed box. The existence of nanoscale Au protrusions around the ND base periphery provide evidence of the extremely strong LPR fields generated by the NDs. It must be noted that the smallest practically achievable lamella thickness was on the order of tens of nanometres (*t* ~30–70 nm) — less than the nanodot diameter, but significantly thicker than the width expected for the CFs.

The STEM images were acquired in transmission mode and therefore, when passing through the CrO_x_ of a CF, the electron beam also interacted with a significantly greater mass of pure Cr_2_O_3_ (see Figure S2). As a result, CFs appear as regions of the oxide with a slightly elevated Cr/O ratio. Further to this issue there are a number of factors which make direct observation of CrO_x_ filaments extremely challenging, particularly their nanometric dimensions and a low probability of an entire CF being confined within a given ND lamella.

Despite these challenges, we were able to resolve a number of candidates for CFs. As expected, they are apparent as subtle variations in the calculated Cr/O map shown in Fig. 2(e). The two highlighted CF filaments contain an elevated Cr content relative to their lateral surroundings and vertically span the oxide barrier. They also display a conic shape (with a diameter of approximately 1–2 nm at either the Au or Cr side) consistent with filament-growth models proposed for other oxides^19, 41^. This tapered cross-section further reduces the detected Cr/O ratio toward that of pure Cr_2_O_3_ (see Figure S2). There are also incomplete filament-like structures observed in the Cr/O map, in which the elevated relative Cr signal is concentrated near one or other metallic layer. We therefore infer that the CF growth process begins from a pure metal layer. For reference, typical EELS spectra for the oxide layer and a CF are presented in Fig. 2(f). We note that filaments are also evident through mapping the integrated L3/L2 ratio for chromium (see Figure S5), with a drop in the ratio value within the CFs. A comparison with literature values reveals that this reduced L3/L2, combined with a subtle shift to lower energy for both the Cr L3 and Cr L2 edges, is consistent with metallic Cr CFs (see Fig. 2(f), S5 and Table S1)^42^.

Figure 2 presents one of the main results of our work: it confirms the presence of CFs in our plasmonic heterostructures. The formation of CFs in Cr_2_O_3_ can be considered as a plasmon field-assisted ion transport process, with the CF described as a sub-stoichiometric CrO_x_ region. Earlier experiments have shown that CF formation arises from the generation of defects in the bulk oxide^43, 44^, a process which could happen due to local dielectric breakdown^45^. Oxygen atoms start to leave their lattice position and drift toward the metallic anode, leaving behind a local conductive path. This work on CrO_x_ is in agreement with prior TEM measurements in amorphous silicon^46^, alumina^47^, and a number of transition metal oxides (TaO_x_, HfO_x_, and TiO_x_)^19^ where it was shown that the host metal cations are mobile in films of 2 nm thickness. Filamentary switching is generally attributed to ion movement, possibly aided by Joule heating and nonlinear high-field kinetics^46–48^. Modelling predicts strongly localised plasmonic fields between Au NDs and the Cr film due to the interaction of each ND with its electromagnetic image^49^. The maximum field occurs very close to the ND, decaying exponentially away from the ND surface. The corresponding field enhancement factor depends on the choice of ND size and periodicity, and in this case is evaluated to be ~100 for an oxide barrier of around 1 nm, a thickness revealed by ellipsometry measurements of our oxidized Cr films (see Figure S1).

The conductive state of CFs connecting NDs with the Cr layer can be assessed by measuring the relative optical transparency of the devices. Relative optical transparency is defined as the ratio of light transmission through the whole structure to light transmission through the Cr layer without NDs thus yielding only the contribution from the NDs. As mentioned above, the relative absorption of the square array in the infrared region (far from the LPR) is given by ${Abs}\approx {\rm{Re}}(\frac{4\pi }{cR})$. When CFs are not formed, the resistance is infinite and the relative transparency goes to 100%. Any deviation of relative transparency from 100% allows one to estimate the resistance of the CFs. Previously we have established the existence of quantized relative transparency (in units of *πα*, where *α* is the fine structure constant) in the studied heterostructures at near infrared wavelengths (*λ* ~1 μm)^24^. Here, the optical transmission of the samples was measured over much broader spectral range (*λ = *0.3–12 μm) in order to provide unambiguous proof that the observed quantization is due to CF shunt currents.

**Figure 3:**
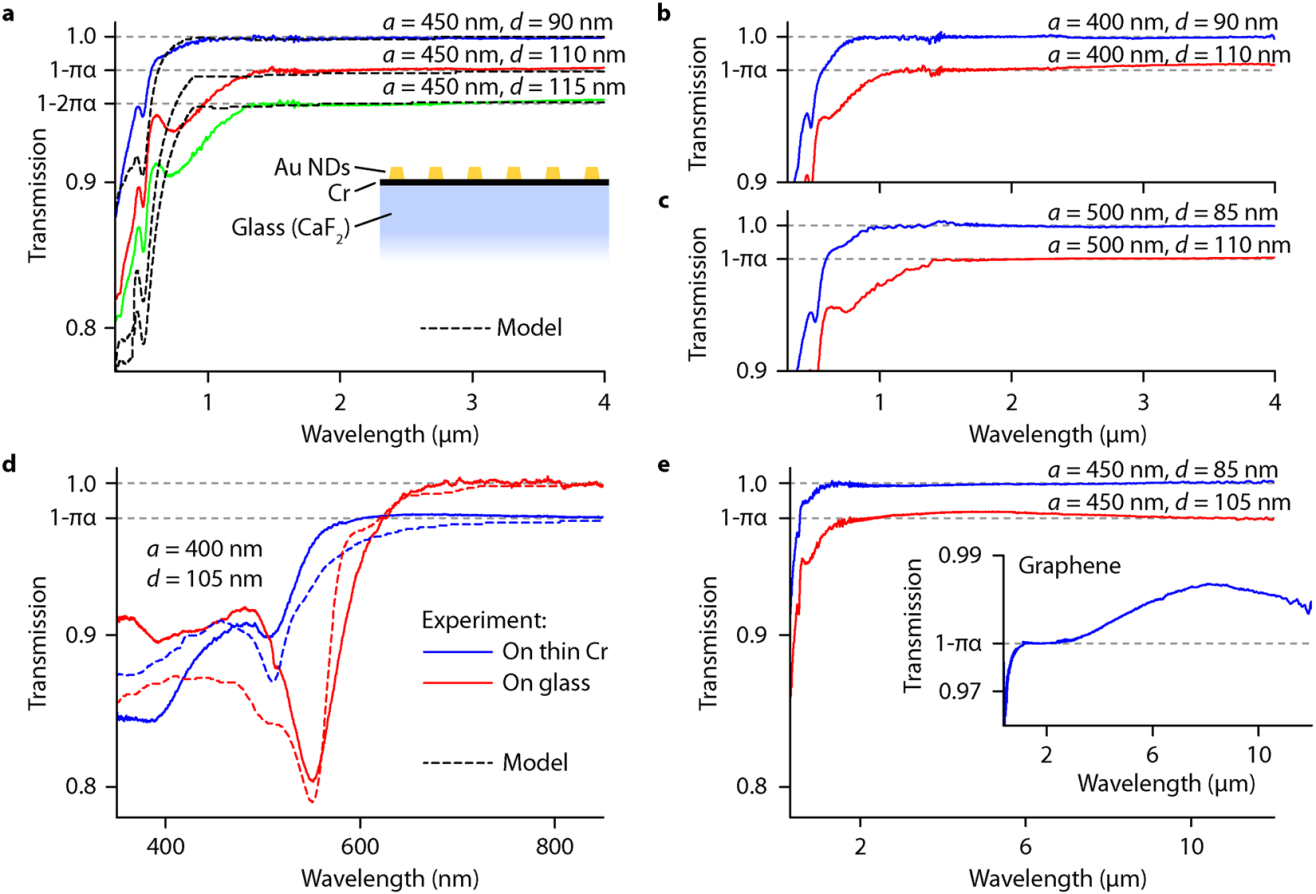
Relative optical transmission spectra of Au ND arrays. (**a**–**c**) Relative transmission spectra measured for different ND sizes and a lattice period, solid lines. The dashed lines in (**a**) show the modelling results for these ND arrays. The inset in (**a**) provides the schematics of samples. (**d**) Suppression of plasmon resonances of arrays fabricated on conductive Cr substrate, solid blue line, and restoration of plasmon resonances after etching the Cr sublayer, solid red line. The dashed lines show the results of modelling. (**e**) The relative transmission spectra for the plasmonic nanoarrays fabricated on a CaF_2_ substrate measured to the mid-infrared region. Inset shows measured transmission spectrum of a monolayer, exfoliated graphene flake.

Figure 3(a–c) show the normal-incidence transmission spectra, normalized by the transmission through the Cr layer nearby, for nanoarrays of various periodicities (*a = *400, 450 and 500 nm) and nanodot diameters (*d* = 85–115 nm) measured in the spectral range of 0.3–4 μm. The infrared transmission of each ND array reaches a well-defined discrete level that depends on the dot size and the lattice constant. These discrete relative optical transparency levels indeed depend solely on universal constants; taking values of *nπα*, where *n* is an integer. Figure 3(e) shows that the quantised relative transmission extends out to the mid-infrared limit of the measurement range at *λ* = 12 μm, well away from the expected LPR of the array (500–700 nm), with no further resonances appearing at longer wavelengths. This (combined with Kramers-Kroning relations) effectively excludes all explanations for the quantized optical transparency based on the dielectric functions of the metals involved and strongly hints towards the explanation based on quantized conductance of CFs. For comparison, the inset of Fig. 3(e) shows the measured transmission spectra of exfoliated graphene. The near-infrared absorbance (*A* = *πα* = 2.3%) of a graphene flake is consistent with previously reported values^50–52^, and is defined by the two-dimensional nature and gapless electronic spectrum of graphene — the high frequency conductivity for Dirac fermions in pristine, defect- and impurity-free graphene is equal to *e* ^2^/4*ħ*. However, at longer wavelengths, the measured transmission of graphene deviates from the predicted value due to a non-zero Fermi level in the graphene sheet leading to a Drude contribution to the absorbance. In our case, the Drude contribution of the Cr sublayer is cancelled due to the fact that we are measuring relative optical transparency.

It is worth noting some other important properties of the measured optical spectra. The presence of the Cr sublayer strongly affects the LPRs of the ND array: it blue-shifts LPRs for smaller dots without CFs and suppresses LPRs completely for larger dots (which grow CFs via the plasmonic mechanism), see Fig. 3(a–c). For comparison, Fig. 3(d) shows the optical spectra of one of the studied arrays before and after the removal of the Cr metallic layer where one can see the reappearance of the LPR at *λ* ≈ 550 nm after Cr removal. In all cases the spectra display an interband scattering peak at *λ = *520 nm which coincides with the LPR for smaller dots. For larger dots (*d* > 100 nm), an additional near-infrared absorption feature (*λ* ~700–800 nm) occurs due to a diffraction-coupled resonance (DCR) at the array-air interface^30, 31^.

In order to model the optical properties of our heterostructures, the optical response of arrays of Au NDs was calculated with a coupled dipole model, using an effective polarizability and an effective dielectric function^53–55^. This basic model was modified to account for electron transport through the CFs. Specifically, we used a quantum-corrected model (QCM)^32, 34, 36, 37^ to calculate the effect of quantum conductance on the optical response of the plasmonic system (see Supplementary Information for modelling details). In brief, within the QCM the tunnelling gap is described by an effective local dielectric function, *ε*_*g*_(*t*) = *ε*_*∞*_ + *i*4*πσ*_*QT*_/ω. Here *ε*_∞_ = 1 and *σ*_*QT*_ describes the current flow through the narrow gap as a consequence of electron tunnelling and is given by *σ*_*QT*_ = *G*/*t*, where *G* is the quantum conductance. According to the TEM and ellipsometric measurements the gap distance was set as *t* = 1 nm. The quantum conductance *G* = *βG*_0_, where *G*_0_ = 2*e* ^2^/*h* is the conductance quantum and *β* is the coefficient arising due to resistance averaging over the dot and the array^24^. The results of these simulations are shown by the dashed lines in Fig. 3(a) and (d). These theoretical results, using conducting CFs, give a good agreement with the measured spectra in terms of the quantized infrared transmission, LPR and DCR suppression, and the strong feature around 520 nm.

In the end, we address three important questions arising with respect to the observed phenomenon. First, why is the quantization of relative optical transparency so accurate (as compared with, e.g., contact resistance measurements)? The answer to this question lies in an extremely large number of nanodots being interrogated by light. Simple estimates show that light beams used in our experiments illuminate around *N* ≈ 10^4^ nanodots improving the statistic accuracy by a factor of $\sqrt{N}\approx 100$. (Making a comparison with contact resistance measurements, a single measurement of optical transmission would correspond statistically to ~10^4^ measurements of a contact resistance.) Second, why is the appearance of fractional quantization numbers so rare? There are three main reasons for this: i) extremely high quality of the studied nanodot arrays: the physical and geometrical parameters of the system (including nanodots) are the same around the whole area of lithography, ii) a strong drop in surface charges and plasmon induced fields after formation of CFs around a nanodot, iii) a very large number of nanodots involved in producing optical signals making deviations and defects statistically insignificant. As a result, in order to observe fractional quantization numbers one needs to fabricate an array with dot sizes and separations such that it would be on the brink of producing a higher quantization number under natural illumination. It is easy to see that this is a statistically rare event. (We had just one sample from ~100 which demonstrated half quantized transparency.) Third, why is the measured quantum of optical conductance *e* ^2^/4*ħ* different from the conductance quantum of 1D filaments 2*e* ^2^/*h*? In our previous work^24^ we explained this difference by averaging the shunt resistance between nanodots over the position of the CF around the nanodot perimeter. In short, if we consider a single dot, the first CF would grow close to the perimeter (when the plasmon fields are the strongest) at a “weak” spot in the dielectric native oxide layer. As soon as the first CF grows, the plasmon charges at the nearest edge of the nanodot will travel into the chromium sublayer and no longer accumulate locally. This implies that the opposite side of the dot would experience much larger plasmon electric fields (not compensated by the opposite charges on the other side of the dot). Hence, an exactly opposite CF will grow. These two CFs suppress plasmon resonance for one in-plane light polarization. To suppress plasmon resonances for the perpendicular light polarization two more opposing CFs will grow. Since an initial weak spot could be at any point close to the dot perimeter, one needs to perform angular averaging which leads to the difference between optical conductance and conductance quantum^24^.

**Figure 4:**
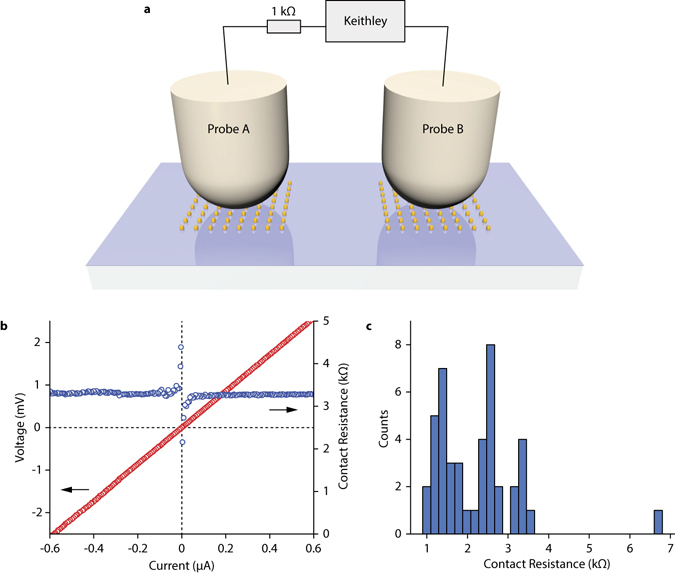
Measurements of shunt resistance between nanodots. (**a**) Schematic of the measurement setup. (**b**) A typical V-I curve and the calculated probe contact resistance measured on two arrays with quantized relative near-infrared transparency. (**c**) Histogram of measured probe contact resistances.

The averaging procedure is quite complicated and might be unpersuasive. For this reason, we decided to obtain independent experimental evidence for its necessity by interrogating the electrical conductance of CFs with the help of direct measurements of the shunt resistance between nanodots. To this end, we performed resistance measurements on two nearby nanodot arrays which shows πα quantization using a standard probe station technique; see Fig. 4(a) and Methods. Figure 4(c) shows the histogram of the measured contact resistances. We see that the peaks in the histogram of the shunt resistance are observed at 6.7 kOhm (close to *R*_*q*_/2 = 6.5 kOhm, where *R*_*q*_ = 12.9 kOhm is the resistance quantum), 3.3 kOhm (close to *R*_*q*_/4 = 3.2 kOhm), 1.4 kOhm (close to *R*_*q*_/8 = 1.6 kOhm) and 2.5 kOhm (close to *R*_*q*_/8 + *R*_*q*_/16 = 2.4 kOhm). This suggests that a typical nanodot is electrically connected to the chromium sublayer by 4 CFs with the quantized resistance corresponding to the resistance of a quantum wire as explained above. Hence, the observed difference between the optical conductivity and the conductivity of a quantum wire could indeed come from angular averaging of the distribution of the CF positions.

## Conclusion

Using transmission electron microscopy, we have experimentally observed conductive filaments with quantized conductance in a thin oxide layer of a plasmonic heterostructure. Growth of filaments is the result of a plasmon-induced electro-chemical redox reaction coupled with oxygen vacancy migration triggered by enhanced plasmonic fields. The filaments have sizes of around a few nanometres and provide shunt currents in a metal sublayer resulting in a quantized conductance and relative optical transparency of the arrays. The quantized infrared transmission enables optical read-out for our devices. The results presented here establish a connection between the plasmon-induced electro-chemistry and quantum transport phenomena, thus bringing a new perspective to the emerging field of quantum plasmonics.

## Methods

### Sample fabrication

Prior to array patterning, a thin (5 nm) Cr layer was deposited on the 1 mm-thick glass (or 1 mm-thick CaF_2_ for measurements at longer wavelengths) substrates to improve subsequent lithography. This continuous, conductive sublayer was not removed in the completed samples. Uniform square arrays of NDs, each covering an area of 200 μm × 200 μm, were patterned using standard electron beam lithography (LEO system). Array periodicities and average dot diameters were varied between samples. A double-layered resist technique was employed to facilitate lift-off (bottom layer: 80 nm of 3% 495 PMMA, top layer: 50 nm of 2% 950 PMMA), developed in 1:3 MIBK:IPA developer for 30 s. Note that the combination of two-layer PMMA resists with thickness of bottom and top layers smaller than the height of Au dots allows us to improve the effectivity of lift-off process and prevents defect formation. A thick (90 nm) Au layer was deposited using an electron-beam evaporator at a base pressure of 5 × 10^−6^ Torr (with a 3 nm Cr layer to enhance adhesion). Finally, samples were immersed in acetone for approximately one hour for metal lift-off. Array dimensions were confirmed using scanning electron microscopy (SEM). Sample fabrication was discussed in depth in previous works^30, 56, 57^.

ND lamella cross sections were prepared using a dual beam focused ion beam (FIB) and SEM instrument (FEI Dual Beam Nova 600i). Prior to FIB milling the ND array is coated with ~10 nm of carbon and ~3 nm of Au/Pd via sputtering. A protective platinum ‘strap’ (~15 × 1 × 1 µm) is then deposited diagonally across the array using a gas-injection system and the electron and ion beams. FIB milling using 30 kV gallium ions is used to cut the resulting lamella free from the substrate using decreasing current steps of 9.3–1 nA. A micromanipulator needle is used to remove the lamella from the trench and transfer it to an Omniprobe copper half grid where it is secured by further Pt deposition. Low energy ion polishing (5 kV and 2 kV at 80 pA) was used to remove side damage and thin the lamella to 30–70 nm thickness (estimated by EELS).

### Optical measurements

A Bruker Vertex 80 Fourier Transform Infrared (FTIR) spectrometer, combined with a Hyperion 3000 microscope, was used to measure polarised infrared transmission spectra. A variety of sources and detectors, combined with aluminium coated reflective optics enable this system to be used from visible to mid-infrared wavelengths (*λ* = 1–12 μm). The entire beam path purged with dry, CO_2_-scrubbed air to minimise strong atmospheric absorption bands. FTIR measurements were complemented by measurements with Ocean Optics USB2000 and USB4000 fiber optic UV/visible/near-infrared grating spectrometers, using a collimated xenon lamp source, focussed to a spot size of ~50 μm. Transmitted light was collected by a 400 μm-diameter multimode fiber placed immediately behind the sample. In both techniques, relative transmission spectra were measured as the ratio of sample transmission to that of the substrate (measured close to the sample and including the metallic sublayer). All measurements were performed under standard room temperature and pressure conditions.

### Structure characterization

High resolution STEM imaging was carried out using a probe side aberration-corrected FEI Titan G2 80–200 kV with an X-FEG electron source operated at 200 kV. Bright field (BF) and high angle annular dark field (HAADF) imaging were performed using a probe convergence angle of 21 mrad, a HAADF inner angle of 48 mrad and a probe current of ~75 pA. Elemental analysis was performed using a probe current of 480 pA, the Titan’s high collection efficiency Super-X energy dispersive x-ray detector system and a Gatan Quantum ER electron energy loss spectrometer. Spectroscopy data was denoised using principal component analysis (PCA) and fitted using two different model-based approaches as implemented in the open-source HyperSpy library^58^, see supporting information. The number of components considered after PCA denoising was estimated to 9 after careful inspecting of the so-called “scree plot” and the loading of the components^59^.

The structural characterization was also done by atomic force microscopy (AFM). AFM analysis is performed in the tapping mode on the sample to examine the surface morphology in several scan areas closed to arrays of gold nanodots. AFM image of the CrO_x_/Cr/glass nanostructure is shown in Fig. S7. We also determined the surface roughness from AFM data. The AFM images showed that the rms value of the surface roughness were in the range of 0.3–0.4 nm suggesting that the films consisted of closely packed nanograins with fine nanograin sizes.

### Contact probe measurements

Electric measurements were performed using a Cascade Microtech PM8 probe station with accurate electric probe positioning (better than 1 µm). Two probe needles (Ni plated steel with tip radii of 30 µm) were used for contacting the gold nanodots. The large size of the tip needles allowed us to reliably secure a contact to a small number of nanodots (as compared to sharper needles with a radius below 1 µm). The second probe was brought into contact with a second array with a relatively large force, with the goal of achieving good Ohmic contact with low resistance (<1 kOhm measured with respect to chromium sublayer). Consequently, several dots are contacted by this second probe. For sample protection, a 1 kOhm resistance was placed in series with the shunt resistance and its value later subtracted from the measured results. The resistance values were obtained from the dc V-I curve measured with a Keithley 2636B source meter. The V-I curves were found to be linear whenever a stable contact was achieved (during the measurements the electric current did not exceed 1 µA). A typical measured V-I curve is given in Fig. 4(b). It is worth noting an existence of a resistance spike observed at small currents. This spike could be connected to a small contact voltage due to different work-functions of electrons or, alternatively, it could be explained by light-induced transfer of hot electrons^60^ due to plasmon resonance. Since we were interested in robust values of the conductive filament resistance (which were obtained at larger values of applied voltages) we decided to leave this interesting topic for future investigations.

## Electronic supplementary material

Supplementary Information
