# Supplementary material for: Plasmon-induced nanoscale quantised conductance filaments
Source: Sci Rep. 2017 Jun 6;7:2878. doi: 10.1038/s41598-017-02976-7 (PMC5460164; doi:10.1038/s41598-017-02976-7)
Supplement: Supplementary file 1 — Supplementary Information [file 41598_2017_2976_MOESM1_ESM.pdf]

Supplementary Information for

Plasmon-induced nanoscale quantised conductance filaments

Vasyl G. Kravets[1], Owen P. Marshall[1], Fred Schedin[2], Francisco J. Rodriguez[1], Alexander A. Zhukov, Ali Gholinia[3], Eric Prestat[3], Sarah J. Haigh[3], and Alexander N. Grigorenko[1]

[1] School of Physics and Astronomy, University of Manchester, Manchester, M13 9PL, UK

[2]National Graphene Institute, Manchester, M13 9PL, UK

[3] School of Materials, University of Manchester, Manchester, M13 9PL, UK

Sample Fabrication and Measurement

All nanodot arrays were fabricated on 1 mm thick, microscope glass (or CaF_2) substrates. Prior to array patterning, a 5 nm thick metallic sublayer of Cr was deposited onto the substrate using a Moorfield electron beam evaporation system at a base pressure of ~10^{-7} mbar and a growth rate of 0.5 nm/s using a target of 99.99% Cr from Kurt J. Lesker. During deposition the film thickness was monitored by a calibrated quartz microbalance. After sublayer deposition, substrates were removed from the vacuum chamber into ambient conditions, leading to the formation of a thin native oxide layer on the Cr surface. At this point ellipsometric measurements were used to confirm the presence and thickness of this oxide layer. These measurements were performed across a wavelength range of 240-1700 nm in a variable angle spectroscopic ellipsometer (VASE) manufactured by J.A. Woollam, using a rotating compensator-analyzer configuration. Data was collected at angles of incidence from 50° to 70°, in 5° increments. A Fresnel multilayered model[1,2] — containing a glass (or CaF_2) substrate, metallic Cr and native oxide (Cr_2O_3) layers — was used to fit the measured ellipsometric spectra. The bare substrate was also measured and modelled using the Cauchy equation. Fitting was performed using the Woollam WVASE32 software, which contains tabulated values for the complex refractive indices of Cr and Cr_2O_3. Typical measured ellipsometric Ψ spectra and fitted model results are shown in Figure S1. Layer thicknesses extracted from fitting to Ψ and Δ (not shown) give values 4.2 nm for Cr and 1.1 nm for Cr_2O_3. Note that the measured (and fitted) oxide thickness was not completely homogeneous across the substrate, 1.1 nm was the modal average of measurements at

multiple sites across the substrates. These layer thicknesses evaluated from optical measurements correlate closely with the values estimated from TEM measurements (see Figure 1, main text). Subsequently, each substrate was patterned with square arrays of Au nanodots (NDs) using electron beam lithography (LEO-RAITH), with array periods ranging from 320 to 500 nm, and dot diameters ranging from 85 to 115 nm.

In order to perform scanning transmission electron microscope (STEM) imaging and analysis, lamellas with a thickness of less than a single ND diameter were prepared. Lamellas were milled and extracted from the arrays using the focussed ion beam (FIB) lift-out technique in an FEI Dual Beam Nova 600i system. FIB lamellas were extracted along an angle which was off-axis with respect to the ND array (see Figure S2(a)), reducing the probability of failing to intersect multiple NDs. A three-dimensional schematic illustrating the geometry of a typical ND lamella cross section is presented in Figure S2(b). The protective Pt layers deposited immediately prior to sample preparation have been omitted for clarity. Note that although the lamellas were typically thinner than the ND diameter ($t < d$), they were much thicker than CrO_x filaments under investigation. Therefore, filaments appear in the TEM measurements as a subtle graduated elevation in the Cr/O ratio, as illustrated in Figure S2(c). High resolution STEM imaging and elemental analysis was performed using an FEI Titan G2 80-200 kV STEM. Elemental mapping was performed on a number of dots using energy dispersive x-ray spectroscopy (EDS) and electron energy loss spectroscopy (EELS) (for details see Methods).

EELS Results and Analysis

The presence of CFs is clearly shown by the Cr/O map of Figure 1(d). Figure S3(a) displays a single experimental spectrum (red dots) extracted from the spectrum image and its corresponding fitted model (blue line) obtained using the HyperSpy library. The model is composed of three components: a power law function to model the background due to inelastic scattering at lower energy and two Hartree-Slater edges to model the O K and the Cr $L_{2,3}$ edge[3]. Each component of the model has been convolved with the EELS low-loss (Figure S3(b)) to account for the plural scattering of the electron beam within the specimen. This low-loss spectrum was acquired quasi-simultaneously at the same position on the specimen. Finally, the Cr/O ratio was determined by integrating the counts in the fitted edges and computing corresponding Hartree-Slater cross-sections. Due to the strong overlap of the O K and Cr $L_{2,3}$ edges this curve fitting approach provides better quantitative results than the standard method to quantify EELS core-loss spectrum, which involves extrapolating the background before the edge using a power-law function and integrating the signal in the edge[4]. In fact, in our case, the O K edge is too close to the Cr $L_{2,3}$ edge and too strong to obtain a reliable background

extrapolation under the Cr $L_{2,3}$ edge[3]. In a solid the fine structure of the core-loss edges are too complicated to model with simple function. An equalisation function can instead be used to approximate the relative density of unoccupied states in the O K and Cr $L_{2,3}$ edges, thereby improving the quantitative accuracy[5].

An in-depth analysis of the Cr $L_{2,3}$ edge fine structure, in particular the so-called "white line" provides further insight into the composition of the CFs. Further fitting was performed on the Cr $L_{2,3}$ edge, using two Hartree-Slater edges to model the transition of the unoccupied states in the continuum and two Gaussian peak functions to describe the characteristic L_3 and L_2 white-line of the Cr L edge[6]. This enabled precise calculation of the integrated L_3/L_2 ratio. The contribution of each component in the fitted model is shown in Figure S4. As with the previous fitting routine each component of the model was convolved with the EELS low-loss to take into account plural scattering, which is important to obtain good fitting accuracy. The resulting L_3/L_2 map obtained from the ratio of the corresponding fitted Gaussian functions is presented in Figure S5(a). CF filaments are evident (albeit slightly less clearly than for the Cr/O map) as regions with lower L_3/L_2 values. Furthermore, the chromium L_3 and L_2 centre energies are lower in the CFs than the surrounding oxide, as shown in the example spectra of Figure S5(b) (spectra taken from Figure 1(e) and normalised to the L_3 peak). Literature L_3/L_2 values, and their individual centre energies are given in Table S1[7]. Considering these values, the measured peak shifts and reduced L_3/L_2 in the CFs are consistent with metallic Cr filaments.

Ellipsometry Results

To better understand the role of LSPRs and collective DCRs in CF formation, the optical properties of ND arrays were studied via ellipsometry at non-zero angles of incidence ($\theta \neq 0^{o}$); at normal incidence the metallic sublayer prevents these resonances from being observed [8,9]. Figures S6(a-c) show the measured ellipsometric parameters Ψ and Δ and the reflectance spectra for various nanoarrays at $\theta_{inc} = 65^{o}$. At large θ_{inc} both types of plasmon resonance, which were absent for normal incidence, reappear. Furthermore, the main LSPR mode ($\lambda_{LSPR} \approx$ 550-600 nm) – which is an out-of-plane non-suppressed mode – tends to blue shift with increasing ND size, whereas the DCR positions remain relatively unchanged. The appearance of such dips is due to coupling of the incident light to out-of-plane (*z*-axis) LSPRs [9,10]. In fact, for certain combinations of a and d, R_p tends toward zero in the range $\lambda \approx$ 550-650 nm for a particular resonant angle of incidence, θ_{res} (close to 65° for the measured samples). The maximum variations in Ψ and Δ also occur at θ_{res}. Crucially, at longer wavelengths the values of Ψ, Δ and R_p tend toward constant values which are insensitive to the ND

size. Simultaneous quantized transmission and constant reflection imply quantized infrared absorption within the nanodot array. In this region, far from the plasmon resonances, the optical properties of the ND array therefore originate from the tunnelling conductivity in the insulating gap. Figures S6 (d)-(f) show modelled ellipsometric spectra. The collective plasmon resonances were determined by diffractive coupling of 100 NDs and the simulation results match well with the experimental data, at least in the region of plasmon excitation. The main resonance peak position blue-shifts with increasing dot size and we are able to achieve a vanishingly small reflectance of green light at θ_{res}. We note that in these simulations the optical spectra are highly sensitivity to the choice of tunnelling conductivity.

Figure S6 (g-i) also shows the spectral dependences of the ellipsometric parameters Ψ and Δ, and *p*-polarized reflectance (R_p) spectra for the same ND arrays presented in Figure S6 (a-c), but in this case a smaller angle of incidence (θ_{inc} = 45°). As is the case for θ_{inc} = 65°, there is a pronounced out-of-plane localized plasmon resonance in the region around λ = 550-650 nm and DCRs for λ = 700-1000 nm. The ellipsometric study, therefore, reveals that the out-of-plane plasmonic resonance of the arrays is not suppressed by the Cr metallic-oxide sublayers illuminated by oblique light and that a plateau exists in the near-infrared region for Ψ and Δ, and R_p, where these parameters tend toward constant values. The R_p spectra contain significant features in vicinity of the LSPR and DCRs, consistent with the Ψ and Δ spectra. In fact, the dip in R_p associated with the DCR is extremely pronounced, dropping almost to zero near λ = 600 nm.

Modelling

To calculate the transmission and reflection of the ND arrays we used the Maxwell-Garnet effective medium approximation (EMA) in order to describe the optical constants of plasmonic layers[11]. In the EMA the effective dielectric function (ε_{eff}) of the nanocomposite material is related to the effective dipole polarizability of the NDs (α_{eff}), and can be written in form of the Clausius-Mossoti equation:

$$\frac{\varepsilon_{eff} - \varepsilon_d}{\varepsilon_{eff} + 2\varepsilon_d} = \frac{f}{r^3}\alpha_{eff} \qquad (1)$$

where ε_d is the permittivity of surrounding dielectric, r is the radius of the (cylindrical) NDs and f is the ND volume filling fraction. This yields the following extended Maxwell-Garnett formula[11]:

$$\varepsilon_{eff} = \frac{r^3 + 2f\alpha_{eff}}{r^3 - f\alpha_{eff}}\varepsilon_d \qquad (2)$$

where the Au NDs possess a polarizability of $\alpha = 4\pi r^3[(\varepsilon - \varepsilon_d) / (\varepsilon - 2\varepsilon_d)]$ — in this work the complex dielectric function of Au is extracted from ellipsometric measurements of a 90 nm thick Au film. This dielectric function can be expressed as a sum of Drude and Lorentz terms:

$$\varepsilon(\omega) = \varepsilon_0 - \frac{\omega_p^2}{\omega^2 + i\omega\gamma} - \sum_{j=1}^{3} \frac{\Delta\varepsilon_j \Omega_j^2}{\omega^2 - \Omega_j^2 + i\omega\Gamma_j} \quad (3)$$

with, in this case, fitting parameters of: $\varepsilon_0 = 3.9$; $\Delta\varepsilon_j = (0.25,0.5,3.75)$; $\hbar\omega_p = 8.97$ eV; $\hbar\gamma = 0.03$ eV; $\hbar\Omega_j = (2.68,3.09,4.35)$ eV; $\hbar\Gamma_j = (0.449,0.845,3)$ eV, where $j = 1,2,3$. In our simulations we also introduce a radiative correction factor, such that the polarizability of the nth dot, $\alpha_n = \alpha / (1 - 2ik^3\alpha / 3)$, where k is the free space wavenumber $(= 2\pi/\lambda)$[12]. The spectral position of the DCRs can be modelled using the coupled dipole approximation (CDA), in which the collective effect of the oscillating dipoles can be described by an effective collective polarizability: $\alpha_{eff} = \alpha_n / (1 - \alpha_n S)$, where S is a term representing the retarded dipole sum[12,13]. DCRs are associated with the poles of α_{eff}. Using Equation 2 we can express the effective dielectric function ε_{eff} in terms of α_{eff}. This dipole approximation is adequate when distances between particles are relatively large ($a > d$), i.e. for small volume fractions f (in our case f~0.1). It was found that the coupled dipole approximation describe DCR behaviour extremely well. An in-depth discussion of these results is beyond the scope of the present work, but will form the basis of a future publication.

Fresnel equations were used to calculate the transmission spectra, at normal incidence, for a three layer system: a uniform layer of Au NDs in air (with an effective medium ε_{eff}), a thin Cr sublayer, and a thick glass substrate (1mm). Our experimental results show that the plasmonic properties of Au ND arrays can be suppressed by a conductive nanobridge across the nanogap between the NDs and the metallic Cr. As suggested previously[14,15], we assume that an excited LSP leads to the ND surface charge localized around the ND perimeter which leads to formation of CFs. In our model we add a quantized conductance to effective polarizability of NDs and calculated optical properties of the arrays using an effective medium approximation described above. We found that electromagnetic theory, modified by a quantum-corrected approach (QCM)[14-17], correctly predicts the optical properties of Au ND arrays.

To model the effect of quantum tunnelling on the optical properties of the ND array we used the QCM approach, modified to account for electron transport through the CF. In this approach, the conductive filament is described using effective local dielectric function (ε_g), obtained from the quantum conductivity (σ_{QT}) across the gap following with separation distance, t, as

$$\varepsilon_g(t) = \varepsilon_\infty + i\frac{\sigma_{QT}(t)}{\varepsilon_0\omega}$$

where t is the lengths of the filament and, in this case, ε_∞ was set equal to 1. Note that σ_{QT}, and thus ε_g are functions of t. The conductivity $\sigma_{QT}(t)$ of the CF is given by[18]: $\sigma_{QT}(t) = \beta G_0/t$, where $G_0 = 2e^2/h = 7.748\times10^{-5}$ S is the quantum of conductance. The coefficient β gives the quantum conductance of a single CF in units of G_0: $G = \beta G_0$.

Figure S1. Ellipsometric spectra of Cr and Cr_2O_3 layers. Experimental Ψ spectra (solid lines) are fitted by a Fresnel model (dashed lines) with thicknesses: substrate (1 mm), Cr (4.2 nm) and Cr_2O_3 (1.1 nm).

Figure S2. Schematic explanation of STEM cross sectional sample geometry. (a) FIB lamellas were extracted from samples at an angle (dashed line) to the array axes in order to ensure that multiple nanodots were sectioned. (b) Illustration of a nanodot within the FIB lamella of thickness, t. The additional protective coating layers deposited immediately prior to FIB milling have been omitted for clarity. Although the lamellas were typically thinner than the nanodot diameter ($t < d$), they were much thicker than the typical CrO_x filaments, which therefore appears in TEM measurements as a subtle graduated elevation in the Cr/O ratio, as illustrated in (c).

Figure S3. Curve fitting of the O K and Cr $L_{2,3}$ edges for the Cr/O map. (a) Experimental and fitted data in a CF and (b) corresponding EELS low-loss spectrum used in the convolution.

Figure S4. Curve fitting of the Cr $L_{2,3}$ edges for the L_3/L_2 map. The model of the fit consists of the sum of a Hartree-Slater edge and a Gaussian function for each edge (L_3 and L_2) convolved with the EELS low-loss to take into account plural scattering of the electron beam in the specimen.

Figure S5. Integrated Cr L_3/L_2 map and peak shift. (a) Lower L_3/L_2 values are observed in CFs. (b) Both the L_3 and L_2 centre values shift to lower energy in CFs. This behaviour is consistent with metallic Cr CFs within a Cr_2O_3 layer.

Figure S6. Ellipsometry spectra of Au ND arrays. (a), (b) and (c) Measured Ψ, Δ and R_p, respectively, for the same ND arrays as presented in Figure 2, measured at an angle of incidence, $\theta_{inc} = 65°$. (d)-(f) Modelled Ψ, Δ and R_p spectra for the same ND arrays (S6 (a)-(c)), respectively. (g)-(i) The ellipsometric parameters Ψ and Δ, and *p*-polarized reflection, R_p, respectively, measured at $\theta_{inc} = 45°$ for various dot sizes. Dependences of Ψ, Δ and R_p tend toward a constant value in the near-infrared, independent of ND size.

Figure S7. Structure characterization. AFM image of CrO_x/Cr film around gold nanodots. The rms roughness values calculated from AFM data are only 0.3-0.4 nm.

Table S1. Literature values for EELS parameters of CrO_2, Cr_2O_3, and metallic Cr. Selected values from T. L. Daulton & B. J. Little, *Ultramicroscopy* **106**, 562-573 (2006).

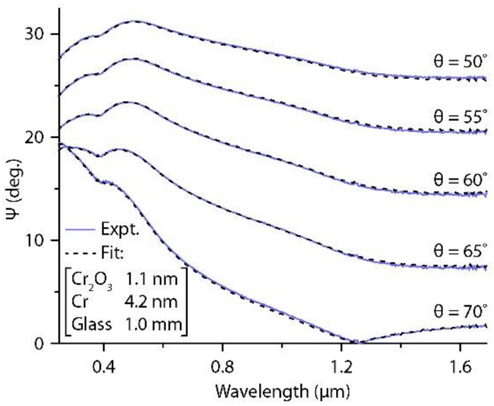

30
20
10
0
Ψ (deg.)
0.4
0.8
1.2
1.6
Wavelength (μm)
θ = 50°
θ = 55°
θ = 60°
θ = 65°
θ = 70°
Expt.
Fit:
Cr2O3 1.1 nm
Cr 4.2 nm
Glass 1.0 mm

Figure S1.

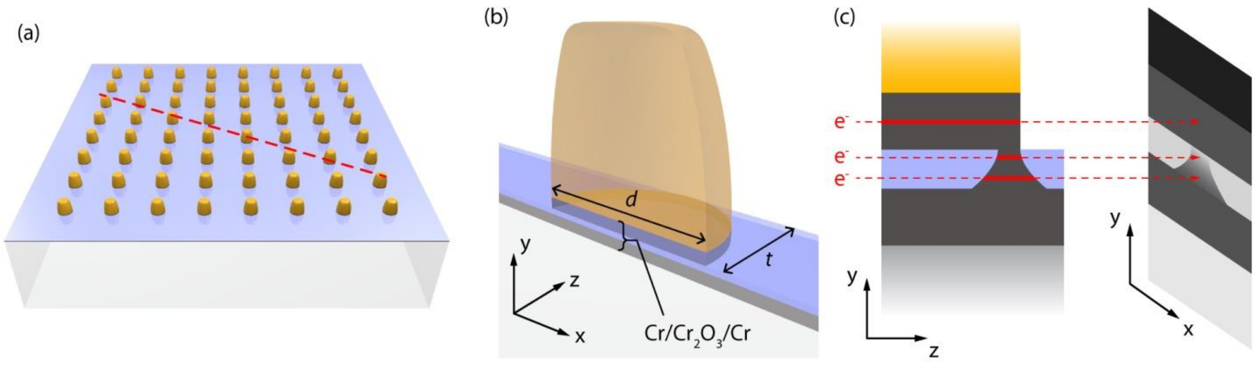

Figure S2.

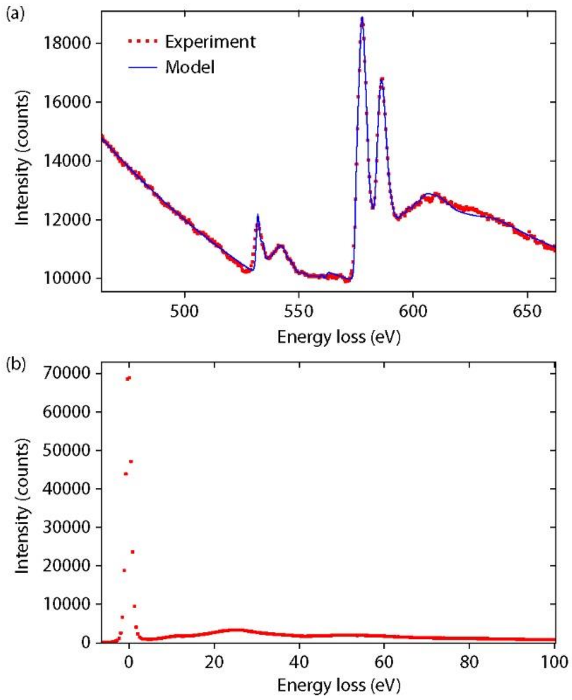
(a)
Experiment
Model
Intensity (counts)
18000
16000
14000
12000
10000
500
550
600
650
Energy loss (eV)
(b)
70000
60000
50000
40000
30000
20000
10000
0
Intensity (counts)
0
20
40
60
80
100
Energy loss (eV)

Figure S3.

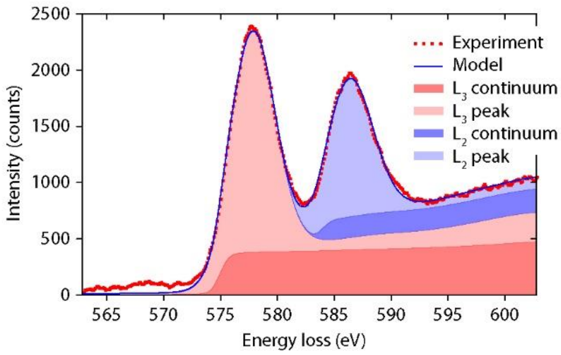
Experiment
Model
L_3 continuum
L_3 peak
L_2 continuum
L_2 peak
Intensity (counts)
Energy loss (eV)
2500
2000
1500
1000
500
0
565
570
575
580
585
590
595
600

Figure S4.

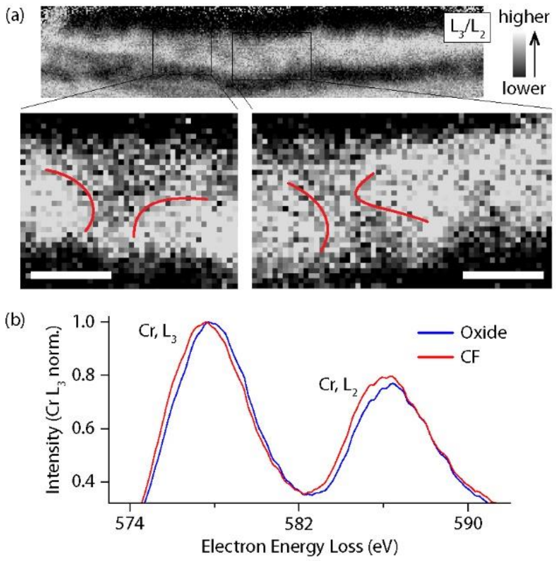
(a)
L_3/L_2
higher
lower
(b)
Cr, L_3
Cr, L_2
Oxide
CF
Intensity (Cr L_3 norm.)
1.0
0.8
0.6
0.4
574
582
590
Electron Energy Loss (eV)

Figure S5.

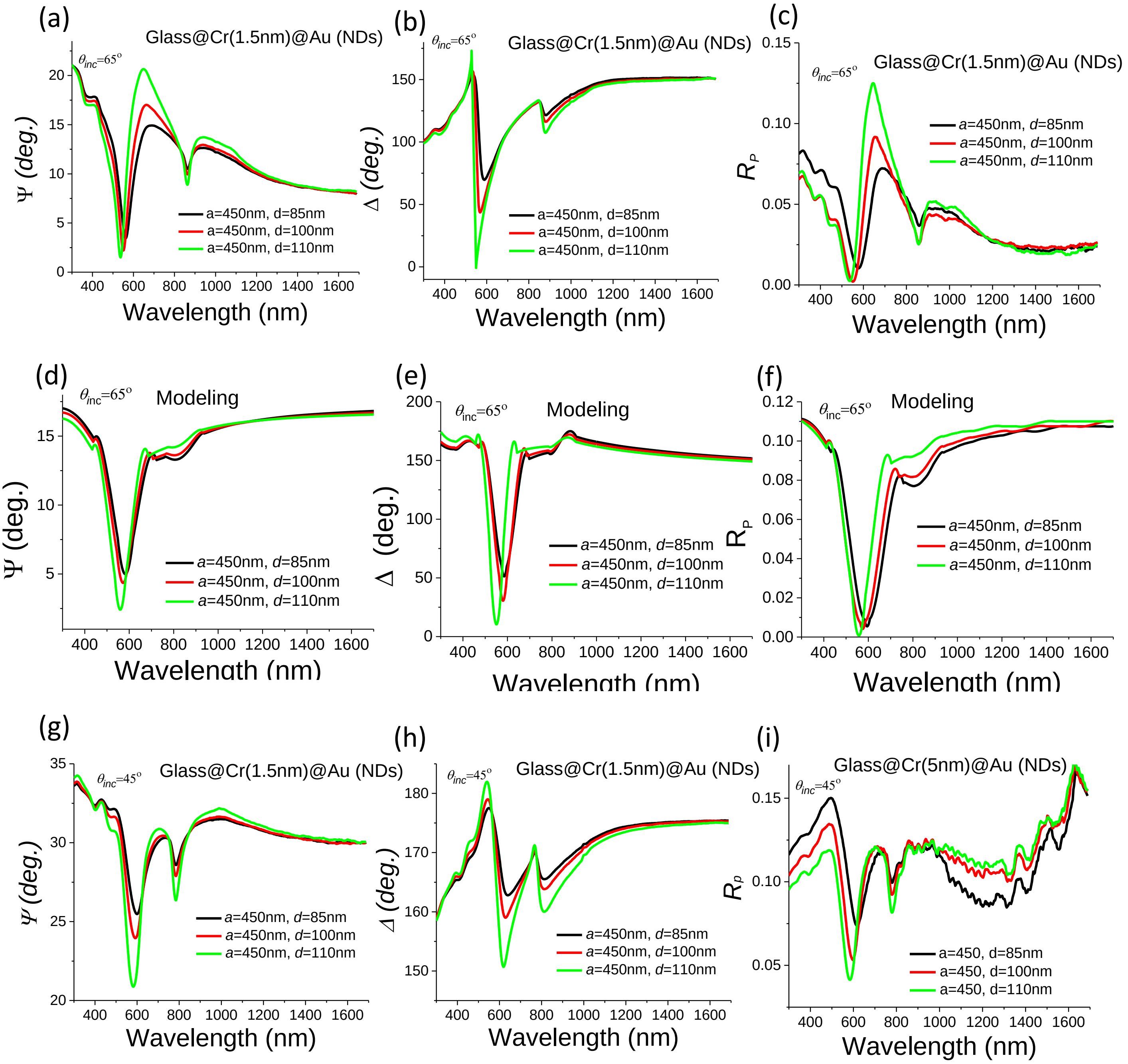

(a)
Glass@Cr(1.5nm)@Au (NDs)
θinc=65°
Ψ (deg.)
a=450nm, d=85nm
a=450nm, d=100nm
a=450nm, d=110nm
Wavelength (nm)
(b)
Glass@Cr(1.5nm)@Au (NDs)
θinc=65°
Δ (deg.)
a=450nm, d=85nm
a=450nm, d=100nm
a=450nm, d=110nm
Wavelength (nm)
(c)
Glass@Cr(1.5nm)@Au (NDs)
θinc=65°
Rp
a=450nm, d=85nm
a=450nm, d=100nm
a=450nm, d=110nm
Wavelength (nm)
(d)
θinc=65°
Modeling
Ψ (deg.)
a=450nm, d=85nm
a=450nm, d=100nm
a=450nm, d=110nm
Wavelength (nm)
(e)
θinc=65°
Modeling
Δ (deg.)
a=450nm, d=85nm
a=450nm, d=100nm
a=450nm, d=110nm
Wavelength (nm)
(f)
θinc=65°
Modeling
Rp
a=450nm, d=85nm
a=450nm, d=100nm
a=450nm, d=110nm
Wavelength (nm)
(g)
Glass@Cr(1.5nm)@Au (NDs)
θinc=45°
Ψ (deg.)
a=450nm, d=85nm
a=450nm, d=100nm
a=450nm, d=110nm
Wavelength (nm)
(h)
Glass@Cr(1.5nm)@Au (NDs)
θinc=45°
Δ (deg.)
a=450nm, d=85nm
a=450nm, d=100nm
a=450nm, d=110nm
Wavelength (nm)
(i)
Glass@Cr(5nm)@Au (NDs)
θinc=45°
Rp
a=450, d=85nm
a=450, d=100nm
a=450, d=110nm
Wavelength (nm)

Figure S6.

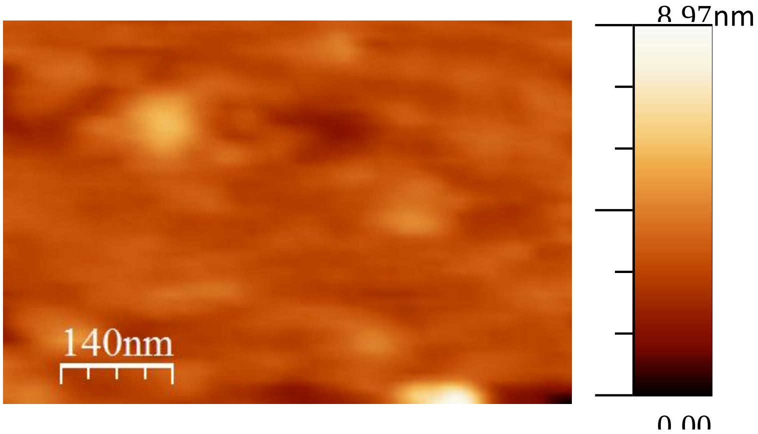

Figure S7.

	CrO_2	Cr_2O_3	Metallic Cr
**Formal valence**	IV	III	0
**L_2 centre (eV)**	587.83 ± 0.03	587.17 ± 0.03	585.79 ± 0.02
**L_3 centre (eV)**	579.70 ± 0.03	578.81 ± 0.03	577.18 ± 0.02
**Integrated L_3/L_2**	1.548 ± 0.002	1.712 ± 0.005	1.525 ± 0.003

Table S1.

References

1 Born, M. & Wolf, E. *Principles of Optics*. 887 (Cambridge University Press, 1999).
2 Azzam, R. M. A. & Bashara, N. M. *Ellipsometry and Polarized Light*. (North-Holland, 1977).
3 Manoubi, T., Tencé, M., Walls, M. G. & Colliex, C. Curve fitting methods for quantitative analysis in electron energy loss spectroscopy. *Microsc. Microanal. Microstruct.* 1, 23-39, doi:10.1051/mmm:019900010102300 (1990).
4 Egerton, R. F. *Electron Energy-Loss Spectroscopy in the Electron Microscope*. (Springer, 2011).
5 Verbeeck, J., Van Aert, S. & Bertoni, G. Model-based quantification of EELS spectra: Including the fine structure. *Ultramicroscopy* 106, 976-980, doi:10.1016/j.ultramic.2006.05.006 (2006).
6 Nord, M., Vullum, P. E., Hallsteinsen, I., Tybell, T. & Holmestad, R. Assessing electron beam sensitivity for SrTiO3 and La0.7Sr0.3MnO3 using Electron Energy Loss Spectroscopy. *Ultramicroscopy*, doi:10.1016/j.ultramic.2016.07.004 (2016).
7 Daulton, T. L. & Little, B. J. Determination of chromium valence over the range Cr(0)-Cr(VI) by electron energy loss spectroscopy. *Ultramicroscopy* 106, 561-573 (2006).
8 Kravets, V., Schedin, F. & Grigorenko, A. Extremely narrow plasmon resonances based on diffraction coupling of localized plasmons in arrays of metallic nanoparticles. *Physical review letters* 101, 087403 (2008).
9 Kravets, V. *et al.* Nanoparticle arrays: From magnetic response to coupled plasmon resonances. *Physical Review B* 90, 125445 (2014).
10 Kravets, V. G., Schedin, F., Taylor, S., Viita, D. & Grigorenko, A. N. Plasmonic resonances in optomagnetic metamaterials based on double dot arrays. *Optics express* 18, 9780-9790 (2010).
11 García-Vidal, F. J., Pitarke, J. M. & Pendry, J. B. Effective Medium Theory of the Optical Properties of Aligned Carbon Nanotubes. *Physical Review Letters* 78, 4289 - 4292, doi:10.1103/PhysRevLett.78.4289 (1997).
12 Markel, V. A. Coupled-dipole approach to scattering of light from a one dimensional periodic dipole structure. *Journal of Modern Optics* 40, 2281 - 2291, doi:10.1080/09500349314552291 (1993).
13 Zou, S. & Schatz, G. C. Narow plasmonic/photonic excitation and scattering line shapes for one and two dimensional silver nanoparticle arrays. *The Journal of Chemical Physics* 121, 12606-12612 (2004).
14 Savage, K. J. *et al.* Revealing the quantum regime in tunnelling plasmonics. *Nature* 491, 574-577, doi:10.1038/nature11653 (2012).
15 Esteban, R., Borisov, A. G., Nordlander, P. & Aizpurua, J. Bridging quantum and classical plasmonics with a quantum-corrected model. *Nature Communication* 3, 825, doi:10.1038/ncomms1806 (2012).
16 Marinica, D. C., Kazansky, A. K., Nordlander, P., Aizpurua, J. & Borisov, A. G. Quantum Plasmonics: Nonlinear Effects in the Field Enhancement of a Plasmonic Nanoparticle Dimer. *Nano Letters* 12, 1333-1339, doi:10.1021/nl300269c (2012).
17 Pérez-González, O. *et al.* Optical Spectroscopy of Conductive Junctions in Plasmonic Cavities. *Nano Letters* 10, 3090-3095, doi:10.1021/nl1017173 (2010).
18 Wen, F. *et al.* Charge Transfer Plasmons: Optical Conductances and Tunable Infrared Resonances. *ACS Nano* 9, 6428-6435 (2015).
